# Bone Strength Measured by Peripheral Quantitative Computed Tomography and the Risk of Nonvertebral Fractures: The Osteoporotic Fractures in Men (MrOS) Study

**DOI:** 10.1002/jbmr.172

**Published:** 2010-06-30

**Authors:** Yahtyng Sheu, Joseph M Zmuda, Robert M Boudreau, Moira A Petit, Kristine E Ensrud, Douglas C Bauer, Christopher L Gordon, Eric S Orwoll, Jane A Cauley, for the Osteoporotic Fractures in Men (MrOS) Research Group

**Affiliations:** 1 Department of Epidemiology, University of PittsburghPittsburgh, PA, USA; 2 School of Kinesiology, Laboratory for Musculoskeletal Health, University of MinnesotaMinneapolis, MN, USA; 3 Center for Chronic Disease Outcomes Research, Veterans Affairs Medical Center & Division of Epidemiology, University of MinnesotaMinneapolis, MN, USA; 4 Department of Epidemiology and Biostatistics, University of California San FranciscoSan Francisco, CA, USA; 5 Department of Radiology, McMaster UniversityHamilton, Ontario, Canada; 6 Bone and Mineral Unit, Oregon Health and Sciences UniversityPortland, OR, USA

**Keywords:** peripheral QCT, men, bone strength, osteoporosis, fracture

## Abstract

Many fractures occur in individuals without osteoporosis defined by areal bone mineral density (aBMD). Inclusion of other aspects of skeletal strength may be useful in identifying at-risk subjects. We used surrogate measures of bone strength at the radius and tibia measured by peripheral quantitative computed tomography (pQCT) to evaluate their relationships with nonvertebral fracture risk. Femoral neck (FN) aBMD, measured by dual-energy X-ray absorptiometry (DXA), also was included. The study population consisted of 1143 white men aged 69+ years with pQCT measures at the radius and tibia from the Minneapolis and Pittsburgh centers of the Osteoporotic Fractures in Men (MrOS) study. Principal-components analysis and Cox proportional-hazards modeling were used to identify 21 of 58 pQCT variables with a major contribution to nonvertebral incident fractures. After a mean 2.9 years of follow-up, 39 fractures occurred. Men without incident fractures had significantly greater bone mineral content, cross-sectional area, and indices of bone strength than those with fractures by pQCT. Every SD decrease in the 18 of 21 pQCT parameters was significantly associated with increased fracture risk (hazard ration ranged from 1.4 to 2.2) independent of age, study site, body mass index (BMI), and FN aBMD. Using area under the receiver operation characteristics curve (*AUC*), the combination of FN aBMD and three radius strength parameters individually increased fracture prediction over FN aBMD alone (*AUC* increased from 0.73 to 0.80). Peripheral bone strength measures are associated with fracture risk and may improve our ability to identify older men at high risk of fracture. © 2011 American Society for Bone and Mineral Research.

## Introduction

Osteoporotic fracture is a global public health concern among older people; they have been linked to increasing mortality, hospitalization, immobility, and dependency. Although women conventionally have been considered at higher risk for osteoporosis and osteoporotic fractures than men, a substantial number of older men do experience osteoporosis,(1) and mortality after a hip fracture is higher in men than in women.(2,3) With increased life expectancy worldwide, the number of hip fractures in men is expected to increase dramatically in the next several decades.(4,5)

Bone mineral density (BMD) measured by dual-energy X-ray absorptiometry (DXA) is currently the “gold standard” used to diagnose osteoporosis and has been shown to strongly predict fractures.(6–8) However, a large proportion of nonosteoporotic women and men suffer fractures.(7,9,10) DXA, a 2D imaging technique, provides integrated areal BMD (aBMD, g/cm^2^) measures that are confounded by individual differences in bone size. Although aBMD is considered a reasonable surrogate measure of bone strength, it does not capture aspects of bone geometry that may contribute to fracture risk, such as bone size, shape, and trabecular and cortical properties of bone. In contrast, 3D techniques of measuring volumetric BMD (vBMD, mg/mm^3^) are not confounded by bone size and also yield separate measures of bone strength and geometry of the trabecular and cortical bone. Research regarding the association between bone strength, as measured by 3D method, and fracture risk has been limited. Cross-sectional studies reported lower quantitative computed tomography (QCT)– or peripheral QCT (pQCT)–derived bone parameters among individuals with fracture than those without fracture,(11–16) and some studies have suggested that these parameters might provide a more in-depth understanding of bone strength and better fracture prediction beyond aBMD.(11–13)

Indeed, the recent American College of Physician guidelines on screening for osteoporosis in men highlighted the need for more research on other BMD screening tests, such as QCT.(17) An earlier report from the Osteoporotic Fracture in Men (MrOS) study found that QCT-derived structural and densitometric measures of the proximal femur predicted future hip fracture, but the ability of QCT to predict fractures was similar to that using traditional femoral neck (FN) aBMD.(18) Central QCT measures are expensive, have a higher radiation dose than peripheral measures, and are limited to hospital settings. Peripheral QCT scanners, on the other hand, are less expensive, impart less radiation exposure, and may be appropriate for clinical research settings. To our knowledge, there are no prospective studies investigating the relationship between pQCT bone parameters and incident fracture in older men. A potential concern for this type of study is that many strength parameters can be derived from pQCT depending on the scanning skeletal site, and the selection of clinically useful parameters can be challenging. The current analysis was designed to objectively select appropriate pQCT bone geometry and strength parameters at the radius and tibia and explore the associations of these bone strength outcomes with incident nonvertebral fracture in a large population cohort of older men enrolled in the Osteoporotic Fractures in Men (MrOS) study.

## Materials and Methods

### Study populations

MrOS is a prospective study designed to identify risk factors associated with osteoporosis and osteoporotic fracture in men. From March 2000 to April 2002, 5995 older men were recruited from six sites across the United States, including Birmingham, AL, Minneapolis, MN, Palo Alto, CA, Pittsburgh, PA, Portland, OR, and San Diego, CA. Details of the study have been published previously.(19,20) In brief, to be eligible for the MrOS study, men needed to be age 65 years and older, be able to walk without assistance from another person, and have had no bilateral hip replacement. From March 2005 to May 2006, active participants were invited to return to the clinic for a follow-up visit. A total of 657 men deceased or terminated before being contacted for the second visit, and fewer than 1% of the men declined to participate. This resulted in a return rate of 98% for the follow-up visit. This analysis only included information from the Minneapolis and Pittsburgh centers due to availability of pQCT scanners. At baseline, both centers recruited 1005 participants, and the numbers were 906 and 886 for the second visit at Minneapolis and Pittsburgh, respectively. A total of 1174 men from both centers received a pQCT scan. After excluding 8 men with missing or invalid information and 23 nonwhite men, this analysis included 1143 subjects. All participants provided informed consent at the baseline and follow-up visits, and the study was approved by the institutional review board at each site.

### Measurements at the follow-up visit

This analysis was performed using data from the follow-up visit. Measurements obtained at the second visit included anthropometry, physical performance, and bone densitometry and body composition by DXA (Hologic QDR-4500W, Bedford, MA, USA). Data collection consisted of demographic characteristics, medical history, medical inventory (both prescription and over-the-counter medications), fracture and fall history, and lifestyle factors by self-administered questionnaire. Body weight was measured in kilograms using balance-beam scales (a digital scale was used at the Portland site). Height was measured in centimeters using a wall-mounted height board. Grip strength was measured twice by a handheld dynamometer (Jamar, Sammons Preston Rolyan, Bolingbrook, IL, USA). Medical history listed in this study was self-reported, and osteoporosis status was defined using male normative values with FN aBMD at the second visit. Medical inventory included both prescription and over-the-counter medications, and each medication was matched to its ingredient(s) based on the Iowa Drug Information Service (IDIS) Drug Vocabulary (College of Pharmacy, University of Iowa, Iowa City, IA, USA).(21)

### Peripheral quantitative computed tomography

A pQCT scan of the radius and tibia was performed using the Stratec XCT-2000 (Pittsburgh site) and the XCT-3000 (Minneapolis site) scanners (Stratec Medizintechnik, Pforzheim, Germany). The only difference between the 2000 and 3000 models is the gantry size. The same acquisition and analysis software was used to analyze scans at both sites. A precision study using a European forearm phantom was performed, and values on the two instruments were similar and within less than 0.5% for total area and from 0.5% to 1.0% for total density.(22) Trained technicians followed a standardized protocol for patient positioning and scanning. A scout view was obtained prior to the pQCT scan to define an anatomic reference line for the relative location of the subsequent scans at the radius and tibia. Tibia length was determined from the medial malleolus to the medial condyle of the tibia, and forearm length was determined from the olecranon to the ulna styloid process. Scans were taken at five different sites: 4% and 33% of the total length of radius and tibia, as well as 66% of the tibia. The scans at the 4% radius and tibia sites represent predominantly trabecular bone, whereas the scans at the 33% and 66% sites represent predominantly cortical bone. A single axial slice of 2.5-mm thickness with a voxel size of 0.5 mm and a speed of 20 mm/s was taken at all locations. Image processing was performed by a single investigator using the Stratec software package (Version 5.5E). Daily phantom scans were analyzed to ensure long-term scanner stability.

### pQCT bone parameters

Parameters measured at all scanning sites of the radius and tibia include total bone mineral content (BMC, mg/mm), total cross-sectional area (CSA, mm^2^), total volumetric bone mineral density (vBMD, mg/cm^3^), and strength-strain index (SSI, mm^3^). At 4% of the radius and tibia, trabecular BMC and vBMD were measured, whereas at the 33% and 66% locations, cortical BMC, vBMD, CSA, periosteal and endosteal circumferences, thickness, cross-sectional and polar moment of inertia (CSMI and PMI), and section modulus (SM, mm^3^) were measured. The formula used to calculate CSMI, SM, and SSI were described in a previous publication by Schoenau and colleagues.(23) CSMI is an estimation of the resistance of bone to bending, whereas PMI represents the ability of bone to resist torsion. CSMI is a function of cross-sectional area and the distribution of bone in that area relative to the axis of rotation. When the bone is distributed further from the axis of rotation, the bone is wider and has more resistance to bending. The SM, an estimator of torsional strength, is derived from the CSMI and the maximum distance between the center of the identified area and its outer boundary. SSI, including both polar (SSIp) and axial (SSIx) measures, is a bending-strength estimator that takes the material properties of bone into consideration by multiplying the SM by the quotient of the measured cortical density and the normal physiologic cortical density (1200 mg/cm^3^). The difference between polar and axial SSI is that SSIp additionally accounts for torsional load. SSIp has been shown to be an accurate and precise indicator of the structural properties of long bones tested in bending.(24) The SSI is more strongly correlated with experimentally determined breaking force than either DXA measures of areal BMD or CSMI or cortical vBMD alone.(24)

### Selection of pQCT parameters

With a total of 58 pQCT parameters measured and limited information regarding the importance of each parameter or scanning site on fractures, a strategy for parameter selection was designed based on principal-components analysis (PCA) and the Cox proportional hazards model in order to limit the number of multiple comparisons. We selected 36 parameters with loading at least 60% (based on varimax rotation) in the first three components that accounted for 71% cumulative variance. These three components individually had eigenvalues greater than 1 and accounted for at least 5% of variance of the data. Within each component, a Cox proportional-hazards regression model [adjusted for age, site, and body mass index (BMI)] with backward elimination was developed to drop parameters whose predicted values could be explained sufficiently by those remaining in the model. A total of 8 variables were selected using these approaches. These variables represented parameters where individuals vary the most and together account for most of the differences between individuals. They are also associated with nonvertebral fracture. Additionally, we included 13 parameters that significantly predicted nonvertebral fractures independent of age, site, BMI, and FN aBMD using separate Cox proportional-hazards regression to avoid the possibility of excluding potentially important parameters from previous step. Table 1 shows parameters included in the current analysis by selection method.

**Table 1:** Selection Methods for pQCT Parameters

Selection methods	Scanning sites	Parameters
PCA and Cox regression with backward elimination^a^	4% tibia	**Total BMC**, trabecular BMC
	33% radius	**Total BMC**, **CSMI, SSIp**
	33% tibia	Cortical BMC, PMI, periosteal circumference
Cox regression^b^	4% tibia	**Total BMC**, SSIx, SSIp
	33% radius	**Total BMC** and CSA, cortical BMC and CSA, periosteal circumference, **CSMI**, PMI, SM, SSIx, **SSIp**
	33% tibia	Total CSA, **periosteal circumference**, SSIx
	66% tibia	SM, SSIp

PCA = principal-components analysis; BMC = bone mineral content; CSA = cross-sectional area; SSIx = sectional stress-strain index; SSIp = polar stress-strain index; CSMI = cross-sectional moment of inertia; PMI = polar moment of inertia; SM = section modulus.

*Note:* Bold = variables appeared in both selection processes.

a Model was adjusted for age, BMI, and site.

b Model was adjusted for age, BMI, site, and FN aBMD.

### Incident nonvertebral fracture

Following the baseline visit, information on self-reported nonvertebral fracture was assessed every 4 months by mail. All reported fractures were centrally reviewed and validated by a physician using radiology reports or operative reports. X-rays were requested and reviewed by a study radiologist if no radiology report was available. Pathologic fractures were excluded, but all other nontraumatic and nonspine fractures after the second visit were included in this analysis.

### Statistical analysis

Characteristics at the second visit and the 21 selected pQCT parameters were compared between individuals with and without an incident fracture using *t* test for continuous variables and chi-square test for dichotomous variables. Cox proportional-hazards regression was used to evaluate the effect of each selected bone parameter on incident fractures, expressed as hazard ratio for nonvertebral fracture per SD decrease in the corresponding bone parameter. Models were adjusted for age, site, BMI, and FN aBMD. We also determined hazard ratios for men in the lower quartiles of pQCT parameters compared with those in the top quartile. Receiver operating characteristics (*ROC*) curves and area under the *ROC* curve (*AUC*) were used to examine the ability of individual pQCT parameters to discriminate nonvertebral fractures, as well as whether the combination of individual pQCT parameters and FN aBMD improved nonvertebral fracture prediction over FN aBMD alone (all models were adjusted for age, BMI, and site). Predicted values from the Cox proportional-hazards regression were used to obtain *AUC*s for the combined effect of pQCT parameter and FN aBMD.

## Results

We compared characteristics for those who returned to the clinic for the second visit by their pQCT status. Men without pQCT measures were older, had lower grip strength, were more likely to have fallen in the past 12 months and to have cancer, were less likely to report excellent/good health, had lower BMIs, and spent less time walking than those who received the pQCT scan (data not shown). Nontraumatic and nonvertebral fractures occurred in 39 participants (3%) during an average of 2.9 ± 0.29 years of follow-up after the pQCT measures were obtained at the second visit. Among the 46 fracture events in 39 of these participants, 60% were hip, ankle/foot/toe, or rib/chest/sternal fractures. Table 2 shows no significant differences in most of the second visit characteristics by fracture status except for grip strength and FN aBMD. Compared to those without fractures, as expected, men with fracture had weaker grip strength and lower aBMD and were more likely to have FN osteoporosis (Table 2). Unadjusted means and percent differences in pQCT parameters between the two groups are shown in Table 3. Compared with men without fractures, pQCT measures were all lower in men who had fracture, with differences ranging from 2.3% to 22.7%, whereas the difference was 11.4% for FN aBMD. All the differences were statistically significant.

**Table 2:** Second Visit Characteristics of Older Men With and Without an Incident Nonvertebral Fracture (Unadjusted)

	No incident fracture (*n* = 1104)	Incident fracture (*n* = 39)
Age (years)	77.2 ± 5.2	78.7 ± 5.4
Weight (kg)	83.9 ± 13.1	83.2 ± 15.9
Height (cm)	173.1 ± 6.8	172.3 ± 7.5
BMI (kg/cm^2^)	27.9 ± 3.8	27.9 ± 4.6
Grip strength (kg)^a^	37.7 ± 7.7	33.4 ± 8.2
Thiazide diuretic	18.7 (206)	12.8 (5)
Vitamin D supplement	61.4 (677)	69.2 (27)
Calcium supplement	25.9 (286)	33.3 (13)
Oral corticosteroids	2.5 (27)	2.6 (1)
Diabetes	15.7 (173)	10.3 (4)
Heart attack	17.1 (189)	15.4 (6)
Stroke	6.7 (74)	12.8 (5)
Hypertension	52.7 (582)	51.3 (20)
Cancer	30.3 (335)	35.9 (14)
Current smoker	2.7 (30)	5.1 (2)
Self-rated good/excellent health compared those with the same age	86.7 (957)	76.9 (30)
DXA density		
FN BMD^a^ (g/cm^2^)	0.79 ± 0.13	0.70 ± 0.13
Average *T*-score^a^	−1.03 ± 0.94	−1.72 ± 0.94
*T*-score category^a^		
Normal	45.4 (500)	18.0 (7)
Low BMD	50.9 (559)	59.0 (23)
Osteoporosis	3.8 (42)	23.1 (9)

*Note:* Values were mean ± SD or mean % (*n*).

a Indicates *p* value comparing second visit characteristics between fractured and nonfractured participants is <.05.

**Table 3:** Unadjusted Means and Percent Differences in pQCT Bone Parameters Between Men With and Without an Incident Nonvertebral Fracture^a^

	No incident fracture (*n* = 1104)	Incident fracture (*n* = 39)	Difference (%)
Tibia 4%			
Total BMC (mg/mm)	378.7 ± 58.3	336.8 ± 70.5	−11.1
Trabecular BMC (mg/mm)	132.3 ± 25.2	120.2 ± 30.2	−9.1
SSIx (mm^3^)	1282.3 ± 371.8	991.1 ± 410.2	−22.7
SSIp (mm^3^)	2426.6 ± 682.9	1890.0 ± 734.2	−22.1
Radius 33%			
Total BMC (mg/mm)	131.3 ± 19.0	116.3 ± 22.5	−11.4
Total CSA (mm^2^)	144.8 ± 19.7	134.4 ± 21.5	−7.2
Cortical BMC (mg/mm)	122.6 ± 19.3	107.6 ± 23.1	−12.2
Cortical CSA (mm^2^)	105.5 ± 15.5	93.2 ± 18.3	−11.6
Periosteal circumference(mm)	42.6 ± 2.9	41.0 ± 3.2	−3.8
CSMI (mm^4^)	1304.6 ± 313.5	1066.6 ± 264.3	−18.2
PMI (mm^4^)	3114.3 ± 777.7	2546.5 ± 677.6	−18.2
SM (mm^3^)	353.7 ± 66.3	310.7 ± 60.7	−12.1
SSIx (mm^3^)	208.8 ± 39.5	177.9 ± 32.3	−14.8
SSIp (mm^3^)	362.2 ± 68.1	320.3 ± 60.6	−11.6
Tibia 33%			
Total CSA (mm^2^)	457.9 ± 51.7	437.6 ± 59.9	−4.4
Cortical BMC (mg/mm)	366.7 ± 45.4	338.6 ± 52.9	−7.7
Periosteal circumference(mm)	75.8 ± 4.2	74.0 ± 5.0	−2.3
PMI (mm^4^)	33012.5 ± 6861.8	30237.4 ± 7980.3	−8.4
SSIx (mm^3^)	1274.1 ± 207.1	1168.7 ± 238.0	−8.3
Tibia 66%			
SM (mm^3^)	3374.1 ± 564.9	2978.2 ± 655.5	−11.7
SSIp (mm^3^)	3372.2 ± 543.3	2988.2 ± 588.7	−11.4

BMC = bone mineral content; CSA = cross-sectional area; SSIx = sectional stress-strain index; SSIp = polar stress-strain index; CSMI = cross-sectional moment of inertia; PMI = polar moment of inertia; SM = section modulus.

a All *p* values < .05.

Table 4 shows the effects of the individual pQCT bone geometry and strength parameters on incident fractures. Most of the pQCT parameters were strongly and significantly associated with fracture risk. Each SD decrease in these parameters was associated with 40% to 120% increased risk of nonvertebral fractures, after adjusting for age, site, BMI, and FN aBMD. Trabecular bone mineral content (BMC) at the 4% tibia and cortical BMC and PMI at the 33% tibia were not significantly associated with incident fracture, where SSIx and SSIp [hazard ratios (HRs) = 2.0 and 1.9, respectively] at 4% of the tibia and CSMI (HR = 2.2), PMI (HR = 2.0), and SSIx (HR = 2.2) at 33% of the radius were among those with the strongest magnitude of association with incident fracture. However, the association with fracture remained strongest for FN aBMD (HR = 2.3/SD decrease).

**Table 4:** Hazard Ratios and *AUC*s for pQCT Bone Parameters and Nonvertebral Fractures in Older Men

	Mean	SD	HR per SD decrease	*AUC*1^a^	*AUC*2^b^
FN aBMD	0.8	0.1	**2.3 (1.6, 3.2)**	0.73	—
Tibia 4%					
Total BMC (mg/mm)	377.2	59.2	**1.7 (1.1, 2.6)**	0.72	0.74
Trabecular BMC (mg/mm)	131.8	25.4	1.2 (0.8, 1.8)	0.67	0.73
SSIx (mm^3^)	1272.0	376.9	**2.0 (1.3, 3.0)**	0.74	0.76
SSIp (mm^3^)	2407.6	681.7	**1.9 (1.2, 2.9)**	0.74	0.76
Radius 33%					
Total BMC (mg/mm)	130.8	19.3	**1.7 (1.2, 2.5)**	0.73	0.77
Total CSA (mm^2^)	144.5	19.9	**1.6 (1.1, 2.2)**	0.69	0.75
Cortical BMC (mg/mm)	122.1	19.6	**1.6 (1.1, 2.4)**	0.73	0.77
Cortical CSA (mm^2^)	105.1	15.7	**1.7 (1.2, 2.5)**	0.74	0.77
Periosteal circumference(mm)	42.5	2.9	**1.6 (1.1, 2.3)**	0.69	0.76
CSMI (mm^4^)	1296.9	314.8	**2.2 (1.4, 3.3)**	0.75	0.80*
PMI (mm^4^)	3095.9	780.9	**2.0 (1.3, 3.1)**	0.74	0.78*
SM	352.3	66.5	**1.7 (1.2, 2.6)**	0.72	0.77**
SSIx (mm^3^)	207.8	39.6	**2.2 (1.4, 3.3)**	0.75	0.79*
SSIp (mm^3^)	360.9	68.2	**1.6 (1.1, 2.5)**	0.70	0.76**
Tibia 33%					
Total CSA (mm^2^)	457.2	52.1	**1.4 (1.0, 2.0)**	0.64^b^	0.73
Cortical BMC (mg/mm)	365.7	45.7	1.4 (1.0, 2.1)	0.70	0.75
Periosteal circumference(mm)	75.7	4.3	**1.4 (1.0, 2.0)**	0.64^b^	0.73
PMI (mm^4^)	32916.9	6917.8	1.3 (0.9, 1.9)	0.64^b^	0.73
SSIx (mm^3^)	1270.5	209.0	**1.4 (1.0, 2.0)**	0.66	0.74
Tibia 66%					
SM (mm^3^)	3360.4	572.5	**1.5 (1.0, 2.2)**	0.73	0.76
SSIp (mm^3^)	3359.0	549.2	**1.6 (1.1, 2.4)**	0.73	0.76

*AUC* = area under the *ROC* curve; BMC = bone mineral content; CSA = cross-sectional area; SSIx = sectional stress-strain index; SSIp = polar stress-strain index; CSMI = cross-sectional moment of inertia; PMI = polar moment of inertia; SM = section modulus.

*Note:* HR models were adjusted for age, BMI, site, and FN aBMD. **Bold** = *p* < .05.

a *AUC*1 for each bone strength parameter (adjusted for age, BMI, and site).

b *AUC*2 for the combined effect of FN aBMD and corresponding bone strength parameter (adjusted for age, BMI, site, and FN aBMD).

* *p* < .05 when compare *AUC* to model with FN aBMD alone.

** .05 < *p* < .1 when compare *AUC* to model with FN aBMD alone.

Discrimination performances determined by *AUC* for FN aBMD and each pQCT parameter are also shown in Table 4. Several pQCT parameters appeared to perform better than FN aBMD, such as CSMI and SSIx at the 33% radius, although the differences did not reach statistical significance. However, when examining whether the addition of individual pQCT measures improved fracture prediction over FN aBMD alone (*AUC* = 0.73), the *AUC*s increased significantly to 0.80, 0.78, and 0.79 (all *p* < .05) for CSMI, PMI, and SSIx at the 33% radius, respectively.

When compared with individuals in the top quartile of pQCT parameters, those in the lowest quartile had 2- to 12-fold greater risk of developing fracture (Table 5). For example, while the HR for the lowest versus highest quartile was 5.1 for FN aBMD, values were 8.0 and 11.7 for PMI and SSIx at the 33% radius, as well was 10.6 for SSIp at the 66% tibia.

**Table 5:** Hazard Ratios (Age-, BMI-, and Site-Adjusted) for pQCT Bone Parameters by Quartile for Nonvertebral Fractures in Older Men

	Quartile 1	Quartile 2	Quartile 3	Quartile 4
FN aBMD	**5.1 (1.9, 13.8)**	1.7 (0.5, 5.1)	0.6 (0.1, 2.5)	Referent
Tibia 4%				
Total BMC (mg/mm)	**4.1 (1.6, 10.2)**	1.2 (0.4, 3.7)	0.5 (0.1, 2.0)	Referent
Trabecular BMC (mg/mm)	**3.8 (1.4, 10.3)**	1.7 (0.5, 5.2)	1.6 (0.5, 4.9)	
SSIx (mm^3^)	**7.8 (2.3, 26.6)**	2.9 (0.8, 11.0)	2.1 (0.5, 8.4)	
SSIp (mm^3^)	**7.6 (2.3, 25.8)**	3.2 (0.9, 11.7)	1.7 (0.4, 7.2)	
Radius 33%				
Total BMC (mg/mm)	**5.4 (2.0, 14.4)**	0.4 (0.1, 2.1)	0.8 (0.2, 3.0)	Referent
Total CSA (mm^2^)	**4.1 (1.5, 11.0)**	1.5 (0.5, 4.8)	1.3 (0.4, 4.3)	
Cortical BMC (mg/mm)	**5.2 (2.0, 14.1)**	0.4 (0.1, 2.1)	0.8 (0.2, 3.1)	
Cortical CSA (mm^2^)	**4.3 (1.7, 10.8)**	0.7 (0.2, 2.4)	0.5 (0.1, 2.1)	
Periosteal circumference(mm)	**4.0 (1.5, 10.8)**	1.5 (0.5, 4.6)	1.2 (0.4, 4.0)	
CSMI (mm^4^)	**5.8 (2.0, 17.3)**	1.8 (0.5, 6.2)	0.8 (0.2, 3.4)	
PMI (mm^4^)	**8.0 (2.4, 27.1)**	2.1 (0.5, 8.6)	1.4 (0.3, 6.2)	
SM (mm^3^)	**7.2 (2.1, 24.6)**	2.1 (0.5, 8.5)	2.0 (0.5, 8.1)	
SSIx (mm^3^)	**11.7 (2.7, 50.9)**	4.2 (0.9, 20.2)	2.0 (0.4, 11.0)	
SSIp (mm^3^)	**4.1 (1.5, 10.9)**	1.2 (0.4, 4.0)	0.8 (0.2, 3.0)	
Tibia 33%				
Total CSA (mm^2^)	2.2 (0.9, 5.1)	1.0 (0.4, 2.8)	0.8 (0.3, 2.2)	Referent
Cortical BMC (mg/mm)	**3.7 (1.3, 10.3)**	2.5 (0.9, 7.1)	0.6 (0.1, 2.6)	
Periosteal circumference(mm)	2.2 (0.9, 5.2)	1.0 (0.4, 2.8)	0.8 (0.3, 2.2)	
PMI (mm^4^)	**2.3 (1.0, 5.3)**	1.1 (0.4, 2.8)	0.6 (0.2, 1.9)	
SSIx (mm^3^)	**4.2 (1.5, 11.2)**	1.0 (0.3, 3.5)	1.6 (0.5, 5.0)	
Tibia 66%				
SM (mm^3^)	**7.8 (2.3, 26.2)**	2.3 (0.6, 9.0)	1.7 (0.4, 7.1)	Referent
SSIp (mm^3^)	**10.6 (2.5, 45.6)**	4.1 (0.9, 19.2)	3.0 (0.6, 14.8)	

BMC = bone mineral content; CSA = cross-sectional area; SSIx = sectional stress-strain index; SSIp = polar stress-strain index; CSMI = cross-sectional moment of inertia; PMI = polar moment of inertia; SM = section modulus.

*Note:* Values are hazard ratios and 95% confidence intervals. **Bold** = *p* < .05.

## Discussion

To our knowledge, this study is first to describe the prospective relationships between pQCT strength parameters and nonvertebral fractures in older men. We found that men with fracture had significantly lower indices of bone strength than those without fractures. Some strength indicators, such as CSMI, PMI, and SSIx at the radius, were significantly associated with incident fractures in three ways: (1) every SD decrease was associated with an approximately 2-fold increase in fracture risk, (2) compared with the top quartile, the lowest quartile was associated with at least 5- to 9-fold higher risk of fracture, and (3) the addition of the individual pQCT strength parameter to models with FN aBMD appeared to increase fracture prediction ability. Although areal measures of BMD are currently considered the “gold standard” to define osteoporosis and determine fracture risk, these measures also have been criticized for their 2D estimation of bone density that does not fully explain bone strength. Emerging research in bone strength and geometry with 3D techniques provides important additional information about skeletal health. However, the most useful geometric and strength parameters to describe fracture risk are not clear due to different techniques applied (ie, pQCT, CT, and MRI), bone outcomes reported, and study designs.

Although bone strength cannot be determined directly, in vivo 3D techniques such as QCT and pQCT provide surrogate measures of bone strength and skeletal geometry. With dedicated software, QCT provides quantitative assessment of CT images beyond visual radiologic evaluation, and QCT-derived parameters (vBMD was reported mostly) have been significantly associated with fractures in both men and women.(13,18,25–27) However, whether QCT measures discriminate fracture better than DXA measures remains unclear. For example, one cross-sectional study reported that QCT vBMD at the spine predicts vertebral fractures better than DXA spine BMD,(26) whereas another study showed no difference in discriminatory power in women.(25) Although QCT has been available for decades, epidemiologic and/or prospective studies of QCT-measured bone parameters and fracture are rare, in part due to the high costs of QCT. In addition, previous studies with QCT bone measures have focused heavily on women and vertebral fractures rather than on the more devastating hip fractures. Black and colleagues reported the first prospective study of hip fracture using central QCT measures of the proximal femur in a large sample of older men.(18) They found strong and inverse relationships between proximal femur QCT-derived BMD and bone volume and hip fracture risk. They observed that individuals in the lowest quartile of these QCT-derived measures were more likely to have an incident hip fracture. Although our study used pQCT parameters of bone strength, we also found a similar relationship between pQCT parameters and nonvertebral fractures. There also appeared to be a threshold effect, where quartiles 2 and 3 were associated with fractures in a much weaker fashion and without statistical significance. In addition, our data suggest that the effects of low bone strength, as measured by pQCT (eg, SSIp and SSIx at the 4% tibia; PMI, SM, and SSIx at the 33% radius; and SSIp at the 66% tibia), on fracture risk may be more profound than that of FN aBMD (HRs ranged 7.2 to 11.7 for pQCT compared with 5.1 for aBMD), but our study is not powered to definitely address this question. Our results suggest that indices of bone strength measured by pQCT may identify men at risk of fracture above and beyond FN aBMD.

The use of pQCT in clinical and epidemiologic research has been limited primarily to skeletal development among children and teenagers due to its sensitivity to growth-related variation. In recent years, there has been growing interest in studying bone strength beyond traditional aBMD, and pQCT is one of the methods used to assess vBMD, bone geometry, and bone strength in adults. The advantage of pQCT includes lower radiation exposure compared to central QCT, relative low cost to operate, easier transportability, and ability to distinguish different bone compartments and skeletal sites that may have different metabolic rates.

Bone strength/geometry parameters measured by pQCT have been associated with vertebral and nonvertebral fractures in vivo,(11,14–16) although most studies were conducted in women.(11,14,15) Mikkola and colleagues reported more negative pQCT features in fractured than nonfractured hip in women.(15) Similar to our findings, studies by Schneider and colleagues(11) and Formica and colleagues(14) found that individuals with fractures had lower or less favorable bone strength/geometry than those without fractures. Our study also showed an 11% difference for FN aBMD between men with and without fracture, which was in line with our results for pQCT parameters (ranged 2% to 22%). This finding is comparable with studies using high-resolution pQCT, where differences in aBMD between fractured and nonfractured females were smaller than most, but not all, geometry and strength parameters.(27–29)

Although some trabecular and cortical bone measures had higher *AUC* values than traditional FN aBMD, the differences did not reach statistical significance, in part due to the small number of fractures in our study. However, the addition of FN aBMD to individual cortical, but not trabecular, pQCT parameters seemed to allow better fracture discrimination than FN aBMD alone. These parameters included CSMI, PMI, and SSIx at the 33% radius site, whereas wrist and arm fractures accounted for only a small portion of the total nonvertebral fracture cases. In contrast, Schneider's group suggested that trabecular BMC and BMD may discriminate fractures better than cortical mass and strength parameters in otherwise healthy women; however, the statistical significance for the differences were unknown, and no aBMD data were compared.(11) Studies comparing fracture discrimination between aBMD and 3D bone measures have shown inconsistent results. Formica and colleagues found that DXA aBMD discriminated fractures better than pQCT parameters in women aged 28 to 84 years,(14) whereas Jamal and colleagues reported opposite findings in hemodialysis (HD) male and female patients aged 50 years and older.(16) A previous study by Black and colleagues found that the combination of three central QCT parameters (trabecular vBMD, percent cortical volume, and minimum cross-sectional area) with FN aBMD measured by DXA did not improve overall hip fracture prediction over FN aBMD alone.(18)

This study has several strengths. MrOS is a well-characterized large study of men who resided in the community at the baseline exam. The availability of both 2D and 3D measures of bone parameters enabled a unique comparison of the relationship of aBMD and pQCT with fracture. In addition, this study used objective criteria to identify the potentially important skeletal parameters in order to avoid excessive multiple comparisons and subjective parameter selection. However, it is also possible that parameters omitted by PCA may play an important role in osteoporotic fracture. To address this issue, we also included parameters that were significantly associated with incident fracture independent of age, BMI, and FN aBMD. Other potential limitations of our study include the small number of fractures, an inability to perform separate analyses of specific types of fractures, insufficient power to stratify the analysis by osteoporosis status, and the limited generalizability of our findings to other populations.

In conclusion, several bone parameters measured by pQCT are strongly associated with nonvertebral fractures in older white men; the risk of fracture was 4 to 9 times higher for individuals in the lowest quartile of pQCT parameters than for those in the highest quatrile. When including DXA measures of FN aBMD in statistical models, three pQCT cortical strength parameters (PMI, CSMI, and SSIx at the 33% radius) had greater *AUC* values for nonvertebral fracture prediction than FN aBMD. Although it is arguable whether the improvement from 0.73 to 0.80 is clinically significant, this study, with only 39 fracture cases, demonstrated the ability of using pQCT strength parameters to predict fractures in older white men. Future studies are needed to understand the role and importance of bone strength and geometry measures on skeletal fragility. Furthermore, it is important to identify most appropriate bone outcomes and measurement technique to effectively determine at-risk men for fracture and monitor the effectiveness of treatment for bone health.

## References

[1] Looker AC, Orwoll ES, Johnston CC (1997). Prevalence of low femoral bone density in older US adults from NHANES III. J Bone Miner Res..

[2] Poor G, Atkinson EJ, O'Fallon WM, Melton LJ (1995). Determinants of reduced survival following hip fractures in men. Clin Orthop Relat Res..

[3] Jacobsen SJ, Goldberg J, Miles TP, Brody JA, Stiers W, Rimm AA (1992). Race and sex differences in mortality following fracture of the hip. Am J Public Health..

[4] Cummings SR, Melton LJ (2002). Epidemiology and outcomes of osteoporotic fractures. Lancet..

[5] Gullberg B, Johnell O, Kanis JA (1997). World-wide projections for hip fracture. Osteoporos Int..

[6] Cummings SR, Black DM, Nevitt MC (1993). Bone density at various sites for prediction of hip fractures. The Study of Osteoporotic Fractures Research Group. Lancet..

[7] Cauley JA, Palermo L, Vogt M (2008). Prevalent vertebral fractures in black women and white women. J Bone Miner Res..

[8] Cummings SR, Cawthon PM, Ensrud KE, Cauley JA, Fink HA, Orwoll ES (2006). BMD and risk of hip and nonvertebral fractures in older men: a prospective study and comparison with older women. J Bone Miner Res..

[9] Wainwright SA, Marshall LM, Ensrud KE (2005). Hip fracture in women without osteoporosis. J Clin Endocrinol Metab..

[10] Schuit SC, van der Klift M, Weel AE (2004). Fracture incidence and association with bone mineral density in elderly men and women: the Rotterdam Study. Bone..

[11] Schneider P, Reiners C, Cointry GR, Capozza RF, Ferretti JL (2001). Bone quality parameters of the distal radius as assessed by pQCT in normal and fractured women. Osteoporos Int..

[12] Andresen R, Haidekker MA, Radmer S, Banzer D (1999). CT determination of bone mineral density and structural investigations on the axial skeleton for estimating the osteoporosis-related fracture risk by means of a risk score. Br J Radiol..

[13] Mackey DC, Eby JG, Harris F (2007). Prediction of clinical non-spine fractures in older black and white men and women with volumetric BMD of the spine and areal BMD of the hip: the Health, Aging, and Body Composition Study*. J Bone Miner Res..

[14] Formica CA, Nieves JW, Cosman F, Garrett P, Lindsay R (1998). Comparative assessment of bone mineral measurements using dual X-ray absorptiometry and peripheral quantitative computed tomography. Osteoporos Int..

[15] Mikkola T, Sipila S, Portegijs E (2007). Impaired geometric properties of tibia in older women with hip fracture history. Osteoporos Int..

[16] Jamal SA, Gilbert J, Gordon C, Bauer DC (2006). Cortical pQCT measures are associated with fractures in dialysis patients. J Bone Miner Res..

[17] Qaseem A, Snow V, Shekelle P, Hopkins R, Forciea MA, Owens DK (2008). Screening for osteoporosis in men: a clinical practice guideline from the American College of Physicians. Ann Intern Med..

[18] Black DM, Bouxsein ML, Marshall LM (2008). Proximal femoral structure and the prediction of hip fracture in men: a large prospective study using quantitative computed tomography. J Bone Miner Res..

[19] Blank JB, Cawthon PM, Carrion-Petersen ML (2005). Overview of recruitment for the osteoporotic fractures in men study (MrOS). Contemp Clin Trials..

[20] Orwoll E, Blank JB, Barrett-Connor E (2005). Design and baseline characteristics of the osteoporotic fractures in men (MrOS) study–a large observational study of the determinants of fracture in older men. Contemp Clin Trials..

[21] Pahor M, Chrischilles EA, Guralnik JM, Brown SL, Wallace RB, Carbonin P (1994). Drug data coding and analysis in epidemiologic studies. Eur J Epidemiol..

[22] Petit MA, Paudel ML, Taylor BC (2009). Bone mass and strength in older men with type 2 diabetes: the osteoporotic fractures in men study. J Bone Miner Res..

[23] Schoenau E, Neu CM, Rauch F, Manz F (2001). The development of bone strength at the proximal radius during childhood and adolescence. J Clin Endocrinol Metab..

[24] Ferretti JL, Capozza RF, Zanchetta JR (1996). Mechanical validation of a tomographic (pQCT) index for noninvasive estimation of rat femur bending strength. Bone..

[25] Lang TF, Guglielmi G, van Kuijk C, De Serio A, Cammisa M, Genant HK (2002). Measurement of bone mineral density at the spine and proximal femur by volumetric quantitative computed tomography and dual-energy X-ray absorptiometry in elderly women with and without vertebral fractures. Bone..

[26] Yu W, Gluer CC, Grampp S (1995). Spinal bone mineral assessment in postmenopausal women: a comparison between dual X-ray absorptiometry and quantitative computed tomography. Osteoporos Int..

[27] Melton LJ, Riggs BL, Keaveny TM (2007). Structural determinants of vertebral fracture risk. J Bone Miner Res..

[28] Vico L, Zouch M, Amirouche A (2008). High-resolution pQCT analysis at the distal radius and tibia discriminates patients with recent wrist and femoral neck fractures. J Bone Miner Res..

[29] Melton LJ, Riggs BL, van Lenthe GH (2007). Contribution of in vivo structural measurements and load/strength ratios to the determination of forearm fracture risk in postmenopausal women. J Bone Miner Res..

